# International Perspectives on Modifications to the Surgical Safety Checklist

**DOI:** 10.1001/jamanetworkopen.2023.17183

**Published:** 2023-06-07

**Authors:** Nathan Turley, Meagan Elam, Mary E. Brindle

**Affiliations:** 1 Department of Surgery, Cumming School of Medicine, University of Calgary, Calgary, Alberta, Canada; 2 Boston University School of Public Health, Boston, Massachusetts; 3 Ariadne Labs, Harvard School of Public Health, Brigham and Women's Hospital, Boston, Massachusetts

## Abstract

**Importance:**

Modification of the World Health Organization’s Surgical Safety Checklist (SSC) is a critical component of its implementation. To facilitate the SSC’s use, it is important to know how surgical teams modify their SSCs, their reasons for making modifications, and the opportunities and challenges teams face in SSC tailoring.

**Objective:**

To study SSC modifications in high-income hospital settings in 5 countries: Australia, Canada, New Zealand, the United States, and the United Kingdom.

**Design, Setting, and Participants:**

This qualitative study used semistructured interviews based on the survey used in the quantitative study. Each interviewee was asked a core set of questions and various follow-up questions based on their survey responses. Interviews were conducted from July 2019 to February 2020 in person and online using teleconferencing software. Surgeons, anesthesiologists, nurses, and hospital administrators from the 5 countries were recruited through a survey and snowball sampling.

**Main Outcomes and Measures:**

Interviewees’ attitudes and perceptions on SSC modifications and their perceived impact on operating rooms.

**Results:**

A total of 51 surgical team members and hospital administrators from the 5 countries were interviewed (37 [75%] with >10 years of service; 28 [55%] women). There were 15 (29%) surgeons, 13 (26%) nurses, 15 (29%) anesthesiologists, and 8 (16%) health administrators. Five themes emerged concerning the awareness and involvement in SSC modifications; reasons for modifications; types of modifications; the outcomes of modifications; and perceived barriers to SSC modifications. Based on the interviews, some SSCs may go many years without being revisited or modified. SSCs are modified to ensure they address local issues and standards of practice and that they are fit for purpose. Modifications are also made following adverse events to reduce the risk of reoccurrence. Interviewees described adding, moving, and removing elements from their SSCs, which increased their sense of ownership in their SSC and participation in its performance. Some barriers to modification included leadership and the SSC’s inclusion in hospitals’ electronic medical record.

**Conclusions and Relevance:**

In this qualitative study of surgical team members and administrators, interviewees described addressing contemporary surgical issues through various SSC modifications. The process of SSC modification may improve team cohesion and buy-in in addition to providing opportunities for teams to improve patient safety.

## Key Points

**Question:**

What do surgical team members and administrators think and experience regarding modifications to the World Health Organization's Surgical Safety Checklist (SSC) in high-income hospital settings?

**Findings:**

In this qualitative of study including 51 participants, 5 themes emerged: awareness and involvement in modifications; reasons, types, and outcomes of modifications; and perceived barriers. Interviews revealed SSCs may go unrevised for many years and face obstacles like leadership and computer system integration, but modifications address local issues, ensure purposefulness, reduce adverse event risk, and foster ownership and participation among health care professionals.

**Meaning:**

These findings suggest that modifying SSCs may enhance patient safety and team cohesion while offering opportunities for increasing team members’ involvement and buy-in.

## Teaser

This qualitative study examines the perspectives of surgical team members and administrators on surgical safety checklist modifications in hospital settings in Australia, Canada, New Zealand, the United Kingdom, and the United States.

## Introduction

Use of the World Health Organization (WHO) Surgical Safety Checklist (SSC) is a standard of surgical practice globally. When implemented well, the SSC can improve outcomes. Its initial pilot study at 8 global sites demonstrated a 36% decrease in postoperative complications and a similar reduction in death rates.^1^

Unfortunately, implementation of the checklist and its effectiveness since the initial pilot study has been variable. Ontario’s rapid rollout in 2010 resulted in a nonsignificant reduction in the risk of death or surgical complications in the setting of little organized implementation.^2^ Conversely, sites using strong and sustained implementation approaches demonstrated outcomes like those of the original pilot study, like the statewide initiative in South Carolina that achieved a 22% reduction in mortality.^3^

Effectively modifying the generic WHO checklist is a critical component of SSC implementation. The SSC was designed to be tailored to address surgical teams’ needs. Effective adaptations increase buy-in and create opportunities to address critical issues affecting the local surgical population.^4^ Conversely, a poorly adapted checklist risks diminishing its value as a communication tool^5^ or failing to address elements that could optimize surgical safety.^6^

The surgical landscape has changed since the introduction of the SSC as have the needs of surgical systems. While still maintaining its fundamental role as a communication tool and memory aid, emerging technology, quality and safety infrastructure, and familiarity with the capabilities of the checklist create opportunities for the checklist to evolve. Understanding how we can effectively modify the checklist creates avenues for improving checklist performance and further awareness of patient safety. In this qualitative study, we explored how teams modified their checklists and the effectiveness of their modifications.

## Methods

### Design

This article focuses on the qualitative group of the first stage in a multiyear project aimed at optimizing clinicians’ use of the SSC. In the first stage, we conducted surveys^6^ and semistructured interviews to explore clinicians’ and administrators’ experiences with the SSC. The current study explores perspectives specific to the motives, approaches, and effectiveness of checklist modifications. The University of Calgary’s Ethics Board approved this study, and the Consolidated Criteria for Reporting Qualitative Research (COREQ) checklist guided its reporting.^7^ All participants provided verbal consent before the interviews began.

### Study Sample

Interviewees comprised administrators and surgical team members in 5 high-income countries: the United States, Canada, the United Kingdom, Australia, and New Zealand. Interviewees were recruited through purposive sampling^8^ of participants from our international survey of checklist users^6^ who indicated interest in being interviewed. We expanded this pool using nonprobability discriminative snowball sampling by asking survey-identified interviewees to suggest colleagues who might be willing to discuss their SSC experiences.^9^ Interview participants were invited by email and were unknown to the study team.

### Data Collection

We conducted semistructured interviews with surgical team members and health administrators from the 5 high-income countries between July 2019 and February 2020 (eAppendix in Supplement 1). Overall, 15 interviews were done in person and 36 were run online, with 33 participants turning their cameras off. Two nonclinical members of the study team, M.E. (a female DrPH student) and N.T. (a male research associate), conducted the interviews. The interviewers are trained in qualitative methods but were previously unfamiliar with the SSC, so aimed to gather extensive information on it from the interviewees. Our interview guide explored (1) current checklist processes, (2) checklist leaders, (3) checklist implementation barriers and facilitators, and (4) checklist effectiveness (eAppendix in Supplement 1). Notes were taken to facilitate analysis. Interviewing stopped at the point of saturation,^10^ ie, when no new information regarding the SSC appeared.^11^ One-on-one interviews lasted approximately an hour and were audio recorded and transcribed. No participants withdrew from the study, and no repeated interviews occurred. Personal information was removed for confidentiality, and participants did not receive transcripts for verification.

### Data Analysis

We inductively coded our qualitative data,^12^ then completed a thematic analysis of it.^13^ A phenomenological interpretive lens was used to explore participants’ perceptions of SSC modifications.^14^ First, M.E. and N.T. collaboratively coded 10 transcripts to ensure consistency in coding definitions and analysis. Regular meetings were held to discuss the coders’ rationale for coding phrases and passages pertinent to SSC modification. Subsequently, the remaining 41 transcripts were coded independently by M.E. and N.T. Through repeated readings of the inductive codes, a thematic analysis was completed to elicit themes from the data,^13^ which the researchers agreed on by consensus.^15^ Any topic concerning SSC modification that was discussed multiple times constituted a theme.^12,16,17^ The context of each piece of coded text was examined to ensure its relevance to SSC modification.^15^ NVivo version 12 was used to organize our study’s analysis (QSR International).

## Results

Between July 2019 and February 2020, we interviewed 51 participants: 15 surgeons (29%), 13 (26%) nurses, 15 (29%) anesthesiologists, and 8 (16%) health administrators, who had firsthand, intimate knowledge of the SSC’s use. For demographics, participants’ representation from the countries was closely distributed (7-12 per country), they primarily had more than 10 years of service (37 [75%]), and, in terms of gender, 28 selected female (55%), 20 selected male (39%), and 3 did not disclose their genders (6%). Most interviewees (45 [88%]) described checklist modifications, and on this topic, 5 themes emerged. The first theme related to the involvement and awareness of surgical team members in the process and purpose of modification. The second encompassed the rationale behind specific SSC modifications. The third comprised types of modifications, and the fourth related to the outcomes of their modifications. The fifth theme entailed barriers faced when considering new modifications to existing checklists. The eFigure in Supplement 1 depicts our themes.

### Awareness and Involvement of Surgical Team in Modifications

Interviewees indicated they used a modified SSC but were often not involved in the modification process—their SSC was an inherited one or institutionally assigned (Table 1). A Canadian surgeon knew their SSC had been modified but was unsure how: “It gets modified. I don’t know if there’s a true, official modified version, or if it’s just an individual modification that happened at the time of implementation.” Health administrators also noted SSC modifications were often made by leaders in isolation. A health administrator from Canada said, “the directives were fairly set in stone as to how people were going to do the Checklist and what was going to be on the Checklist. And, so, the consultative process probably was not as robust, or as contributory, as it could have been or should have been.”

**Table 1.** Awareness and Involvement of Surgical Team in SSC Modifications

Participant	Quotation
Health administrator from the UK	“The modification was done before I started working there.”
Surgeon from Australia	Interviewer: “Now, has the version on the checklist that you’re using changed since its original introduction?”
	Respondent: “Not that I’m aware of.”
Surgeon from Canada	“It gets modified. I don’t know if there’s a true, official modified version, or if it’s just an individual modification that happened at the time of implementation.”
Health administrator from Canada	Regarding involvement in SSC modifications, this interviewee said, “The directives were fairly set in stone as to how people were going to do the Checklist and what was going to be on the Checklist. And, so, the consultative process probably was not as robust, or as contributory as it could have been or should have been.” She then said their SSC was instituted 6 or 7 years ago through a top-down mandate: “It was rather imposed upon everybody and [the administration] said, ‘You’ll do it or else.’”

Abbreviation: SSC, Surgical Safety Checklist.

### Reasons for Specific Modifications

A second theme was participants’ reasons for checklist modifications (Table 2). While most interviewees were not involved in their checklists’ modifications, they shared their understandings of the rationale underlying the modifications. This theme had 2 subthemes: modifications to meet contextual demands and modifications undertaken in response to adverse events.

**Table 2.** Reasons for Specific Modifications

Participant	Quotation
**Context**	
Local issues	
Anesthesiologist from Australia	This interviewee described their SSC as including, “appropriate radiology, appropriate implants, tissue bank or whatever.” They said, “Because of the nature of the work we do, we’ve got some specific things within the checklist.”
Local standards of practice	
Health administrator from the US	This interviewee reported their SSC follows “Florida standards of practice, which lays out who has to be involved in the timeout process.” This administrator added that their SSC includes specimen counts because the state of Florida requires them.
**Ensuring fit for purpose**	
Health administrator from Canada	“We did a much shorter, abbreviated version of the Checklist. So, we adapted our Checklist based on the needs of the patient and on the team’s assessment.”
Surgeon from New Zealand	“We made it what we thought was specific to us. [We] tried to make it as simple as possible, as logical as possible, as consistent as possible, across everything.”
**Adverse events**	
Surgeon from Canada	“When there’s a crisis, like two or three instances of wrong-side surgery in a row, then it creates a more urgent review.”
Anesthesiologist from the UK	This interviewee said their team added SSC items as soon “as they have had an incident or problems.”
Anesthesiologist from Australia	“We were having a lot of trouble motivating people to do a block timeout, and really it was on the back of adverse events [that we added this element to our SSC].”

Abbreviation: SSC, Surgical Safety Checklist.

#### Contextual Demands

Contextual demands consisted of 3 subthemes: local issues, local standards of practice, and modifying the SSC to ensure fit for purpose. First, local issues drove many SSC modifications mentioned by interviewees, like catering to specific surgical procedures at their center. An anesthesiologist from Australia described their SSC as including “appropriate radiology, appropriate implants, tissue bank or whatever…. Because of the nature of the work we do, we’ve got some specific things within the checklist.” Second, several interviewees indicated their teams modified their SSC to reflect local standards of practice and to ensure compliance with safety measures. An anesthesiologist from Australia reported his region adopted a “stop before you block” practice and incorporated it into their SSC to ensure compliance. Third, interviewees described making the SSC fit for purpose by removing items they felt were inapplicable to their surgical situation. A health administrator from Canada said, “We did a much shorter, abbreviated version of the Checklist. So, we adapted our Checklist based on the needs of the patient and on the team’s assessment.”

#### Adverse Events

Adverse events were another reason participants modified their SSCs. Adverse events encompassed issues like close calls, never events like wrong-side surgery, and postoperative complications. A surgeon from Canada said, “When there’s a crisis, like two or three instances of wrong-side surgery in a row, then it creates a more urgent review [of the SSC].” Similarly, a UK anesthetist described items being added as soon “as they have had an incident or problems.”

### Types of Modifications Made by Surgical Teams

Our third theme was modification types, which comprised 3 subthemes related to content and format modifications: adding, moving, and removing elements from the SSC (Table 3). In terms of adding elements to the SSC, a health administrator from the United States reflected, “It’s not exactly what the WHO Checklist was, but it’s almost all the same points and a couple extra, actually, in addition to that.” She indicated surgical staff in her health region “don’t just ask about antibiotic prophylaxis, we also ask about prophylaxis for DVT [deep vein thrombosis].” Other additions included foreseen challenges during the procedure and staff having the chance to express their comfort level, a key consideration for recovery section, and adding nerve block time-outs.

**Table 3.** Types of SSC Modifications Made by Surgical Teams

Participant	Quotation
**Adding SSC elements**	
Health administrator from the US	This interviewee described her SSC as being “not exactly what the WHO Checklist was, but it’s almost all of the same points and a couple extra, actually, in addition to that.” She said her surgical staff “don’t just ask about antibiotic prophylaxis, we also ask about prophylaxis for DVT.”
Nurse and clinical educator from the US	This interviewee said her SSC removed the checkboxes and added bullet points. She said this communicated to surgical staff that SSC items are “not an option, this is mandatory” and “You will confirm your patient, you will use two identifiers.”
**Moving SSC elements**	
Anesthesiologist from New Zealand	Moved the blood loss check to the time-out section of the SSC because “you’re more likely to get surgical involvement at time-out, and they’d probably have a better idea of things like estimated blood loss, given they’re surgeons.”
Anesthesiologist from the UK	Moved the blood loss check to the team huddle before each case because doing it once the patient is anaesthetized is “too late” and “it would take at least an hour cross-matched.”
Nurse from Canada	This interviewee felt the patient-specific concerns question on the SSC should be moved to its sign-in section. She said, “To me, that should not be discussed when the surgeon’s holding the scalpel. That should be discussed when the patient comes in the room.” The “before skin incision” column, said the nurse, is “a little too late.”
**Removing SSC elements**	
Anesthesiologist from New Zealand	On SSC items, this interviewee said, “We took out ones that were clearly inappropriate and in fact a little bit offensive to people. So, saying, ‘Has the anaesthetic machine been checked in this environment?’”
Health Administrator from the US	This interviewee said her health system did away with the checkboxes and replaced them with bullet points to signal “you are to do this.”

Abbreviations: DVT, deep vein thrombosis; SSC, Surgical Safety Checklist; WHO, World Health Organization.

As for moving elements between the checklist’s 3 pause points, interviewees provided examples related to discussing checklist items when the right members are present and appropriate actions can be taken. A New Zealand anesthesiologist said their team moved discussion of blood loss to the time-out because “you’re more likely to get surgical involvement at time-out, and they’d probably have a better idea of things like estimated blood loss, given they’re surgeons.” A UK anesthesiologist, however, said their team moved the blood loss requirements to their team’s huddle before each case.

Regarding removing items, an anesthesiologist from New Zealand said, “We took out ones that were clearly inappropriate and in fact a little bit offensive to people. So, saying, ‘Has the anaesthetic machine been checked in this environment?’” The interviewee expressed there was an individual assigned to checking the anesthetic machine, so the team removed the question, as they felt it was redundant.

### Impact of Checklist Modifications

The impact of modifications on the interviewees’ use of the SSC and surgical experience was our fourth theme (Table 4). Participants who modified their SSCs often noted an improved sense of ownership of the tool and engagement in its use. A UK anesthesiologist said, “It’s very difficult to get engagement” when an organization is “trying to force people to have a standard checklist, [where] probably not all elements will be applicable to each different type of surgery.” Checklist modification “enables local ownership, which, I think, is really good.” Similarly, a health care administrator from New Zealand said surgeons were able to modify the SSC to “suit their place” and “they’ve got buy-in from surgeons because they have input into what it looks like.” After SSC modification, they said, “I think they do take ownership for it then.” Likewise, a nurse and clinical educator from the United States argued that her team’s SSC modifications “brought heightened awareness to what we do” since “we put thousands of patients every year through surgery very safely, and we want to keep it that way.”

**Table 4.** Impact of SSC Modifications

Participant	Quotation
Anesthesiologist from the UK	A modified checklist “Enables local ownership, which I think is really good.”
Health care administrator from New Zealand	Modifications of the SSC enabled it to “suit their place” and “They’ve got buy-in from surgeons because they have input into what it looks like.” These modifications have led the surgeons to “take ownership” of the SSC.
Anesthesiologist from New Zealand	“I’ve seen improved engagement, especially by the surgeons over the years. The nurses were actually quite good adopters, and I think it’s variable amongst the anesthesiologists.”
Nurse and clinical educator from the US	Modifications “brought heightened awareness to what we do.” She said, “We put thousands of patients every year through surgery very safely, and we want to keep it that way.”
Surgeon from New Zealand	Changing the format of their SSC from a paper to a poster made “people feel more involved and engaged, and more ownership of their parts in it.”

Abbreviation: SSC, Surgical Safety Checklist.

### Barriers to Considering Modifications of Existing Checklists

Our discussions with surgical staff also identified institutional barriers to customization (Table 5). One was related to leadership’s desire to modify and the other to the practicalities of modifying after integration into the electronic medical record (EMR). “Everyone is literally expected to adhere to this particular WHO checklist,” said a surgeon from Canada. That SSC version was “simply imposed upon us, and we were told that this is a standard to which all of us must adhere.” Similarly, a surgeon from Australia described his team’s transition from a paper to electronic SSC. With this system, the customization process is lengthy and complicated. He complained, “You can’t bring in what appears to be a successful version [of the SSC] and plug it in.”

**Table 5.** Institutional Barriers to Considering Modifications of Existing SSCs

Participant	Quotation
Surgeon from Canada	“Everyone is literally expected to adhere to this particular WHO checklist.”
	Their version of the SSC was “simply imposed upon us and we were told that this is a standard to which all of us must adhere.”
Surgeon from Australia	On his health system’s electronic health record: “You can’t bring in what appears to be a successful version [of the SSC] and plug it in.”

Abbreviation: SSC, Surgical Safety Checklist.

## Discussion

The interviews in this study suggested that many surgical teams continue to use the WHO SSC without modification for years, which could lead to a lack of engagement and ownership. We can consider how teams might, once again, modify their checklists to address contemporary contextual needs and new clinical issues. Further, our interviewees suggested that reengaging teams in modifying their checklists facilitates improved checklist performance and teams’ awareness of safety issues.

Five major themes emerged from our interviews. First, modifications of the SSC occurred at first introduction, but contemporary staff may not have been engaged in this process, so they may lack knowledge on what changes were made and their rationale. Second, SSC customizations often addressed contextual needs or responded to adverse events. Third, the customizations primarily constituted adding, moving, or removing elements to and from the SSC. Fourth, modification affected team dynamics through additional staff engagement and improved sense of SSC ownership. Finally, some administrative barriers must be considered when revisiting the checklist.

Although our interviews reveal potential benefits of SSC modification for team buy-in and surgical safety, contemporary users may be absent in their SSC’s modification. Performing team-based modifications introduces new team members to the checklist and the rationale for its implementation. This teamwork ensures contemporary needs and contextual considerations are addressed and encourages team members’ ownership of the checklist process. Furthermore, new team members’ SSC perspectives may benefit existing members.^18^

Modifications to the checklist are frequently made to fit context and in response to adverse events. Based on our interviews, we found that fitting the SSC to teams’ contexts involved adding checks required by health systems and tweaking it, so it reflected the nature of the surgical teams’ work. As for other catalysts, adverse events prompted teams to introduce items and checks to prevent further troubles. These observations align with the WHO’s vision for the SSC: that it may be tailored to meet the needs of clinicians and their contexts.

However, participants’ mixed experiences suggest a tension between situational demands calling for SSC revisions and perceived institutional resistance. This suggests institutions should facilitate changes to the SSC and empower surgical teams to make these changes. This empowerment could cast the SSC in a positive light, encouraging meaningful use and potentially preventing adverse events that stimulate reactionary SSCs.

For teams contemplating SSC modifications, various types of changes can be considered. Surgical teams may identify an activity that they believe should be performed as standard practice and integrate it as a content item in the SSC to simplify workflow and encourage compliance. Plus, when considering modifications, teams may identify complex procedures (ie, robotic surgery) or complex patients (ie, neonates) where additional processes need to be considered and important information needs to be communicated. For example, by creating a time-out focused on indications for the cesarean deliveries, the SSC customized by Govindappagari et al^19^ increased communication and agreement between health care professionals on the procedure from 59% to 80%.

Teams should be cautious about introducing changes to the SSC that undermine its role as a communication tool^5^ or make performance so arduous that engagement wanes. Teams should also consider their SSC modality, as the integration of the SSC into EMRs and other electronic tools may foster dysfunction in the operating room by downgrading the SSC’s performance to a tick-box exercise.^20^ Teams should be mindful of the implications for adding or removing items.^5,21^ New items should be evaluated for importance and whether they address the issues the checklist is suitable for. Redundant or widely adopted items, like the pulse oximeter check, may be removed, but items promoting critical communication between team members should be retained.

Thought should also be given to how the SSC will be performed. Many interviewees customized their checklist to specify which people should attend each aspect of the SSC’s performance. This consideration reflects the notion that the presence of certain team members in discussions is essential to ensure the safest surgery.

Although there were institutional barriers, interviewees noted some benefits to modifying the checklist. Modifying the checklist was identified as an opportunity to bring surgical teams together with a shared goal to improve patient safety. Interviewees described the importance of team building and collaboration that resulted from engaging multiple teammates in customizing their SSC. This inclusive behavior reflects contemporary surgical suites and mirrors surgery as being part of a multimodal and multiprofessional care pathway.

One product of staff involvement in the SSC’s customization is a sense of ownership over its use. When pathways and processes are developed with all stakeholders involved, change is more likely accepted.^22,23^ As a New Zealand health administrator said, “People having an opportunity to participate in [the SSC’s] creation was very important, because people just don’t like being told to do something greatly. They’d much rather have ownership.”

The surgical landscape has changed considerably over the last decade. Novel opportunities exist for checklist redevelopment that were not previously considered. The COVID-19 pandemic changed the way surgical care was performed in the setting of widespread, airborne infections, so opportunities to integrate a response into the SSC materialized.^24^ The overprescription of opioids after surgery is a significant contributor to the opioid crisis, with roots in perioperative pain strategies.^25^ As a multiprofessional communication tool, the SSC could address narcotic-limiting pain management strategies just before surgery.

### Limitations

This study has some limitations. First, its focus on 5 high-income countries may not be representative of surgical teams worldwide. Second, more clinicians than health administrators were interviewed, so interpretations may be biased toward checklist users. Third, the study slightly skews toward participants from Australia and New Zealand, and most participants from New Zealand stemmed from snowball sampling, which may promote interviewing like-minded individuals. Furthermore, interviews present a snapshot of participants’ perspectives, which can change. Nonetheless, our qualitative study offers valuable insight into individuals’ perspectives on SSC modification.

## Conclusions

In this qualitative study of clinicians and administrators from 5 high-income countries, participants shared that their SSCs were frequently modified, but contemporary users were often alienated from the modification process. The interviews revealed that local needs and previous adverse events sparked SSC modifications, where teams added, moved, and removed checklist items. The process of modification brought teams together, which influenced their SSC use and work in the operating room. Finally, while barriers may exist, there are significant opportunities for teams to collaborate on improving patient safety by revisiting their current needs and processes and updating their SSCs.

## Notes

Supplement 1. **eAppendix**. Interview Guide **eFigure.** Tree Diagram of Themes and Subthemes

Supplement 2.
Data Sharing Statement
