# Supplementary material for: International Perspectives on Modifications to the Surgical Safety Checklist
Source: JAMA Netw Open. 2023 Jun 7;6(6):e2317183. doi: 10.1001/jamanetworkopen.2023.17183 (PMC10248735; doi:10.1001/jamanetworkopen.2023.17183)
Supplement: Supplement 1. — eAppendix. Interview Guide eFigure. Tree Diagram of Themes and Subthemes [file jamanetwopen-e2317183-s001.pdf]

Supplemental Online Content

Turley N, Elam M, Brindle ME. International perspectives on modifications to the Surgical Safety Checklist. *JAMA Netw Open.* 2023;6(6):e2317183. doi:10.1001/jamanetworkopen.2023.17183

eAppendix. Interview Guide
eFigure. Tree Diagram of Themes and Subthemes

This supplemental material has been provided by the authors to give readers additional information about their work.

© 2023 Turley N et al. *JAMA Network Open.*

eAppendix. Interview Guide

SSC 2.0 Interview Guide - Study ID

[START RECORDER - to capture informed consent interaction]

Introduction

Thank you for agreeing to talk with me today. You have been selected to speak to us because you have been identified as [for clinicians: a clinician who is a member of a perioperative surgical team that uses the Surgical Safety Checklist OR for administrators: a healthcare administrator involved in the implementation or operationalization of the Checklist process at your institution or organization].

Our research project is focused on learning about your use of the World Health Organization Surgical Safety Checklist, any modifications you have made to the checklist, its implementation at your institution, and how to optimize the Checklist's effectiveness toward patient safety.

Your participation in this study is purely voluntary and you may stop interviewing at any point, or refuse to respond to any questions you don't feel comfortable answering. All information will be kept confidential and all results will be reported in the aggregate. Your participation in this interview will not be compensated. We are scheduled to talk for one hour. Does that still work in your schedule?

We will record this interview so that we can transcribe your words accurately. Do you agree to participate in the interview?

PARTICIPANT INFORMATION

Hospital Name: ___________________________________

Interviewee Name and Title: ______________________________

Interviewer(s):

PARTICIPANT DEMOGRAPHICS

[If RECRUITED VIA SURVEY: confirm demographic information provided in the survey]

[If NOT RECRUITED VIA SURVEY, proceed with the following questions]

What is your age: (<=30, 31-40, 41-50, 51-60, 61-70, >70)

You are a: (Clinician on the perioperative team/Health administrator)

Years of experience:

Time worked at current site:

Interview Questions

Probes are *italicized* throughout the document.

Background of SSC, its content, and suggestions for revisions

1. From your perspective, what is the purpose of the WHO checklist?

General Use

2. Thank you for providing us with a copy of the checklist being used at your institution.

[For those recruited through survey]: On your survey, you mentioned that [SURGICAL ROLE] typically leads the process for completing the checklist. Has this changed? Who else is involved?
Why do you think that [ROLE] *should lead the process?*

[For those NOT recruited through survey]: Who usually leads the process to complete the checklist? Who else is involved? Who do you think should lead this process?

© 2023 Turley N et al. *JAMA Network Open.*

Why should this person lead the process?

3. Can you talk me through the process for using this checklist at your institution?

What's the process for participating in the checklist during surgical team changes (e.g., shift changes, lunch breaks, etc.)?

4. **[For surgical team members]:** Has the version of the checklist you're using changed since its original introduction?

If so, what changes have been made and why were they introduced?
From your perspective, how have these changes impacted your team's experience with the checklist?

[For administrators]: To what extent has the checklist been modified for use in your institution? Who led the modification process? Who else was involved?
Why were these modifications made? To what extent do you feel they've been effective?

Implementation

5. To what extent did you receive education or training on the purpose and use of the checklist?

How many trainings received? How often?
What was helpful about the training/education? What could have been done differently?

[If NOT recruited through survey]: What other resources *(e.g., hardcopy materials, direct observation/feedback, etc.)* were used to promote the checklist?
Which of these resources is most impactful?
What was the process for implementing the checklist across the institution? For example, who was the champion (leader), how long did the process take, and what were some improvements you think could be made?
Was there a checklist champion or team that facilitated the implementation of the checklist? Was the champion an administrator or clinician? Were they in a senior role? Had they lead other quality improvement interventions in the past? Was there an incentive to serving in this role?
How long did it take for the checklist to be fully implemented into hospital processes?
PUSH--*Are there areas of improvement for the current implementation activities? What could be done differently?*

6. What challenges were experienced or are ongoing in the implementation of the checklist?

7. In what ways, if any, did you/your institution utilize the WHO or Ariadne Labs implementation manual for the checklist?

We'd like to collect the best practices for introducing and providing training on the checklist and present them to the public to accompany the revised version of the Checklist. What would be the best way to present these (e.g., manual, slide deck)?

Facilitators to Use

© 2023 Turley N et al. *JAMA Network Open.*

8. **[For those recruited through survey]:** Can you tell me more about the In-person educational sessions (e.g., Grand Rounds Lecture) AND Team-based training (e.g., Low and High-Fi Simulation) that you have at your institution *(e.g., number of education/training sessions, type of hardcopy materials, frequency of observation/feedback, etc.)*? Why were these the most useful in promoting checklist use?

9. Is there anything else that facilitates checklist use here?

[For those NOT recruited through survey]: What facilitates checklist use at this institution?
a. Which specific resources (*e.g., in-person or online training, hardcopy materials, direct observation and feedback, etc.*) are more helpful in promoting checklist use? *(Probe for use on specific answers given- e.g., number of education/training sessions, type of hardcopy materials, frequency of observation/feedback, etc.)*
How does institution leadership promote continued use of the checklist?
Are there any current policies or incentives for change that affect checklist use?

Barriers to Use

10. What are some barriers to checklist use at this institution?

Was there resistance to using the checklist? Who was most resistant?
Has anything been done to address these difficulties?
What suggestion do you have for reducing these difficulties?

Effectiveness and Outcomes of SSC

11. What do you consider to be a measure of checklist effectiveness? In your opinion, has the checklist been effective here? Why or why not?

To what extent are checklist use and/or effectiveness monitored at your institution?

12. What item(s) do you feel has the most impact on your patients?

Lessons Learned for Future Use (Implementation, and Sustainability)

13. Looking at the checklist your team is using and reflecting on its use, what changes could be made?

Can you describe any gaps in surgical care that could be addressed with the checklist?
What changes to the checklist would improve its effectiveness? For example, what questions should be added?
What are your thoughts on its current visual layout and formatting (e.g. font size, color scheme, general look/legibility)?

Thank you for your feedback on that—we may circulate a draft of our revised version of the checklist for feedback. Would you be interested in being contacted for reviewing and providing feedback on a draft of the next version?

© 2023 Turley N et al. *JAMA Network Open.*

14. What can be done to tie the checklist into patient safety practices at your institution?

Secondary Questions (ONLY IF EXTRA TIME):

15. What have been some unintended consequences from using the checklist? They can be positive or negative.
16. What's the impact on the SSC on communication? *Only needs to be asked if not mentioned in the beginning or in previous answers.*

Wrap-Up

1. We've reached the end of the interview. **What haven't I asked you that you'd like to tell me?** Any final words?

Thank you for taking the time to meet for an interview. Your feedback is greatly appreciated! If you have any questions or think of anything else you'd like to add to the information you've provided today, please feel free to contact us.
For snowball sampling, ask if interviewee knows of another person that may be willing to speak with us about their experiences with the SSC. Tell them we're especially interested in people who have had negative experiences with the checklist. If they are willing to provide a name, ask them if they'd be willing to introduce you via email (we can compose the email for them if they'd like).

© 2023 Turley N et al. *JAMA Network Open.*

eFigure 1. Tree Diagram of Themes and Subthemes

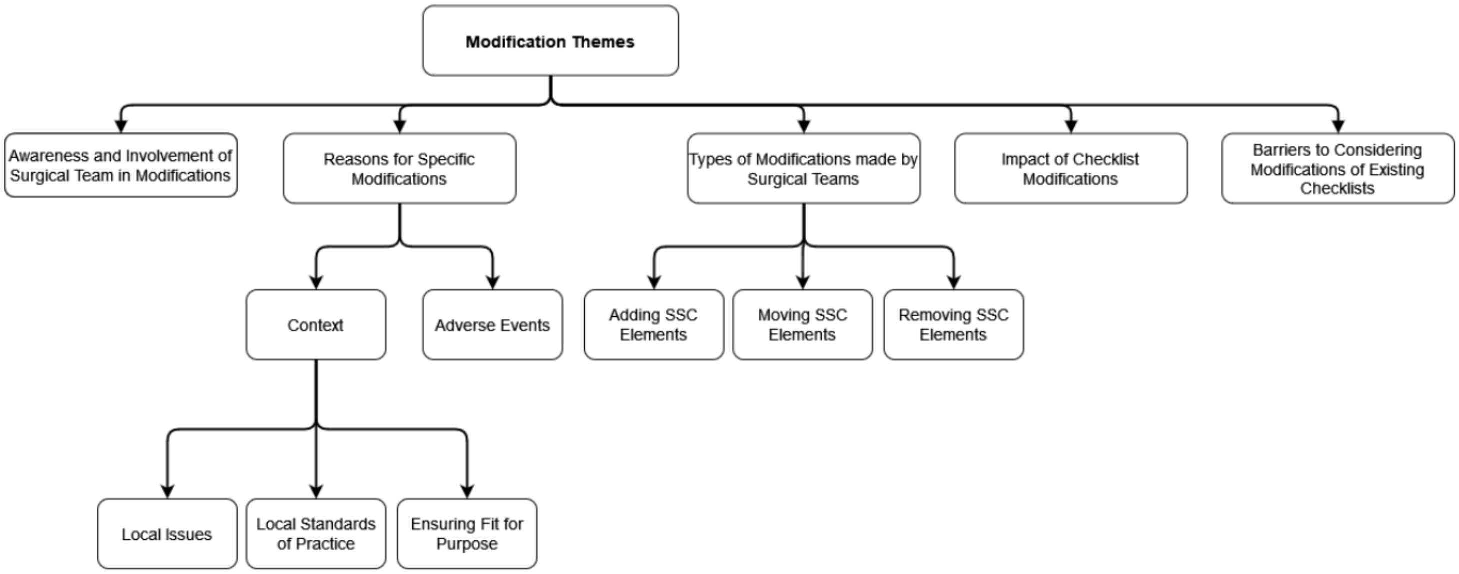

© 2023 Turley N et al. *JAMA Network Open.*
