# Supplementary material for: International Perspectives on Modifications to the Surgical Safety Checklist
Source: JAMA Netw Open. 2023 Jun 7;6(6):e2317183. doi: 10.1001/jamanetworkopen.2023.17183 (PMC10248735; doi:10.1001/jamanetworkopen.2023.17183)
Supplement: Supplement 2. — Data Sharing Statement [file jamanetwopen-e2317183-s002.pdf]

Data Sharing Statement

Turley. International Perspectives on Modifications to the Surgical Safety Checklist. *JAMA Netw Open*. Published June 07, 2023. doi:10.1001/jamanetworkopen.2023.17183

Data
Data available: Yes
Data types: Deidentified participant data
How to access data: By data request only mbrindle@ariadnelabs.org
When available: With publication

Supporting Documents
Document types: Informed consent form
How to access documents: By data request only mbrindle@ariadnelabs.org
When available: With publication

Additional Information
Who can access the data: Data will be made available to researchers whose proposed use of the data has been approved.
Types of analyses: Data will be made available to researchers only
Mechanisms of data availability: Data will be available after approval of a proposal
